# SOX9 Knockout Induces Polyploidy and Changes Sensitivity to Tumor Treatment Strategies in a Chondrosarcoma Cell Line

**DOI:** 10.3390/ijms21207627

**Published:** 2020-10-15

**Authors:** Sabine Stöckl, Georg Lindner, Shushan Li, Philipp Schuster, Sebastian Haferkamp, Ferdinand Wagner, Peter M. Prodinger, Gabriele Multhoff, Melanie Boxberg, Axel Hillmann, Richard J. Bauer, Susanne Grässel

**Affiliations:** 1 Department of Orthopaedic Surgery, Experimental Orthopaedics, Centre for Medical Biotechnology (ZMB/Biopark 1), University of Regensburg, 93053 Regensburg, Germany; Shushan.Li@stud.uni-regensburg.de (S.L.); Susanne.Graessel@klinik.uni-regensburg.de (S.G.); 2 Institute of Microbiology and Hygiene, University Hospital Regensburg, 93053 Regensburg, Germany; Georg1.Lindner@klinik.uni-regensburg.de (G.L.); Philipp1.Schuster@klinik.uni-regensburg.de (P.S.); 3 Department of Dermatology, University Medical Center Regensburg, 93053 Regensburg, Germany; Sebastian.Haferkamp@klinik.uni-regensburg.de; 4 Department of Pediatric Surgery, Dr. von Haunersche’s Children’s Hospital, LMU, 80337 Munich, Germany; Ferdinand.Wagner@med.uni-muenchen.de; 5 Department of Orthopaedic Surgery, Campus Großhadern, LMU, 81377 Munich, Germany; 6 Department of Orthopaedic Surgery, Klinikum Rechts der Isar, Technical University of Munich (TUM), 81675 Munich, Germany; Peter.Prodinger@khagatharied.de; 7 Department of Trauma Surgery and Orthopaedics, Krankenhaus Agatharied, 83734 Hausham, Germany; 8 Center for Translational Cancer Research (TranslaTUM), Radiation Immuno Oncology Group, Klinikum Rechts der Isar, Technical University of Munich (TUM), 81675 Munich, Germany; gabriele.multhoff@tum.de; 9 Department of Pathology, Technical University of Munich (TMU), 80333 Munich, Germany; melanie.boxberg@tum.de; 10 Department of Sarcomas and Musculoskeletal Tumors, Barmherzige Brüder Hospital, 93049 Regensburg, Germany; Axel.Hillmann@barmherzige-regensburg.de; 11 Department of Oral and Maxillofacial Surgery, Center for Medical Biotechnology, University Hospital Regensburg, 93053 Regensburg, Germany; richard.bauer@ukr.de

**Keywords:** SOX9, transcription factor, chondrosarcoma, polyploidy, MMP13, CRISPR/Cas9

## Abstract

As most chemotherapeutic drugs are ineffective in the treatment of chondrosarcoma, we studied the expression pattern and function of SOX9, the master transcription factor for chondrogenesis, in chondrosarcoma, to understand the basic molecular principles needed for engineering new targeted therapies. Our study shows an increase in SOX9 expression in chondrosarcoma compared to normal cartilage, but a decrease when the tumors are finally defined as dedifferentiated chondrosarcoma (DDCS). In DDCS, SOX9 is almost completely absent in the non-chondroid, dedifferentiated compartments. CRISPR/Cas9-mediated knockout of SOX9 in a human chondrosarcoma cell line (HTB94) results in reduced proliferation, clonogenicity and migration, accompanied by an inability to activate MMP13. In contrast, adhesion, apoptosis and polyploidy formation are favored after SOX9 deletion, probably involving BCL2 and survivin. The siRNA-mediated SOX9 knockdown partially confirmed these results, suggesting the need for a certain SOX9 threshold for particular cancer-related events. To increase the efficacy of chondrosarcoma therapies, potential therapeutic approaches were analyzed in SOX9 knockout cells. Here, we found an increased impact of doxorubicin, but a reduced sensitivity for oncolytic virus treatment. Our observations present novel insight into the role of SOX9 in chondrosarcoma biology and could thereby help to overcome the obstacle of drug resistance and limited therapy options.

## 1. Introduction

Chondrosarcoma are known to be the second most frequent form of primary bone sarcoma and typically affect the long bones and pelvis [1]. They are generally thought to be relatively resistant to chemotherapy and radiation due to their high content of extracellular matrix, their small percentage of dividing cells and their low vascularity. Wide surgical excision is still the most effective treatment, but in some cases, sufficient resection is not always possible due to the size, localization or metastasizing status of the tumors [2]. Understanding the detailed molecular mechanism of these tumors may provide new treatment targets and might thereby improve the survival rate of patients with chondrosarcoma [3]. The majority (>85%) of conventional chondrosarcomas consist of primary central chondrosarcomas, which are referred to as such due to their central location in the medullary cavity. A small proportion of conventional chondrosarcomas emerge from the bone surface. In most cases, they develop as a result of a malignant transformation in the cartilage cap of an already existing osteochondroma and are therefore termed secondary peripheral chondrosarcomas [4]. In terms of histology, central and peripheral chondrosarcomas are quite similar. Three different grades (I–III) are defined and used as predictors of clinical behavior [5], which can also be supported by analyzing the nuclear DNA content [6]. Dedifferentiated chondrosarcomas (DDCS) are defined as a highly malignant form of chondrosarcoma and have a particularly poor prognosis with 40–85% metastasis rates. They typically show two different histopathological components: a chondrosarcoma or a well differentiated benign chondral lesion directly flanking a high-grade, non-cartilaginous and malignant compound [7,8].

SOX9 (Sex-determining region Y (SRY)-box 9) is the main transcription factor for chondrogenic differentiation during embryogenesis and has a critical role in triggering the expression of chondrogenic genes. Moreover, Sox9 is crucial for the deposition of extracellular matrix (ECM), which consists in cartilage mainly of type II collagen [9] and large proteoglycans. For the normal development and differentiation of mesenchymal stem cells into chondrocytes, a precise timing of induction and the silencing of SOX9 expression are required. Disturbing this regulation implies a high risk of tumor development as SOX9 is involved as an oncogene in various types of cancer, but it can also act as a tumor suppressor in other tumor entities [10,11]. One possible explanation for the versatility of SOX9 could be a combination of post-transcriptional modifications, the type of tissue in which it is expressed and certain binding partners. To date, only very few studies have been published on the detailed role of SOX9 during chondrosarcoma development and progression [12]. The latest studies emphasize the direct crosstalk between SOX9 and a mutation of isocitrate dehydrogenase (IDH) 1. *IDH1* mutations are found in ~50% of all chondrosarcoma patients and are involved in malignant transformation [12]. Thereby, the association between SOX9 and IDH1 highlight the crucial role of SOX9 in chondrosarcoma biology. The objectives of our research study were to determine the expression pattern of SOX9 in chondrosarcoma biopsies from different grades and thus to understand the role of SOX9 during the process of chondrosarcoma development, progression and dedifferentiation. The analysis of chondrosarcoma cells after the knockdown and knockout of SOX9 was performed to determine the subsequent molecular impact on the carcinogenic properties, downstream targets of SOX9 and therapeutic resistance mechanisms.

## 2. Results

### 2.1. Increased SOX9 Gene Expression in G1, G2 and G3 Chondrosarcoma, but Not in Dedifferentiated Chondrosarcoma

**Figure 1:**
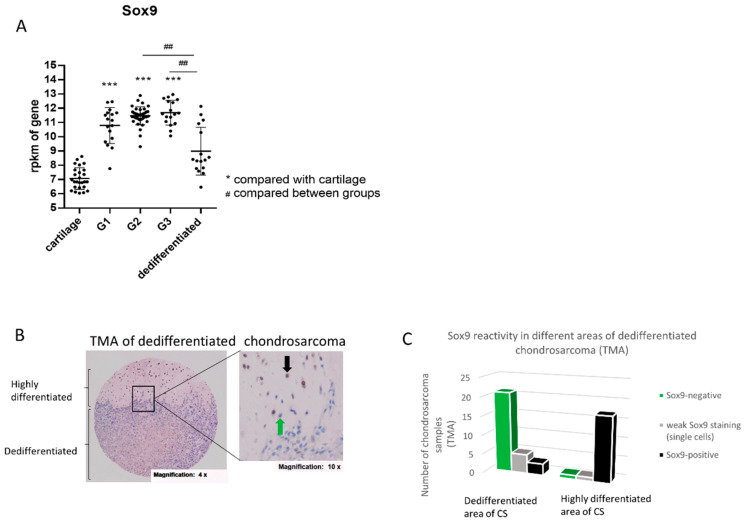
SOX9 expression in the chondrosarcoma samples. (**A**) Meta-analysis of *SOX9* gene expression in human chondrosarcoma with different grades (G1, G2, G3) and in dedifferentiated chondrosarcoma, in comparison to *SOX9* gene expression in healthy cartilage (from non-tumorous cartilage tissue). (**B**) Immunohistochemical (IHC) staining of SOX9 was performed on a tissue-micro-array (TMA) containing dedifferentiated chondrosarcoma. Representative image of nuclear staining intensity for SOX9 in chondrosarcoma tissue is shown. The black arrow indicates a SOX9-positive cell and the green arrow indicates a SOX9-negative cell. (**C**) Distribution of SOX9 reactivity in different areas of dedifferentiated chondrosarcoma samples in a TMA reveals a majority of SOX9-negative samples (21/29) in the dedifferentiated part of the tumor tissue and a majority of SOX9-positive samples (17/19) in the highly differentiated samples. rpkm = reads per kilo base per million mapped reads; G = grade; CS = chondrosarcoma; ## *p* ≤ 0,01; *** *p* ≤ 0.001.

To assess the expression of *SOX9* in chondrosarcoma, we performed a meta-analysis using different open source databases. Thereby, we compared *SOX9* gene expression in healthy cartilage (26 samples) with the expression in grade I (G1) chondrosarcoma (17 samples), grade II (G2) chondrosarcoma (39 samples), grade III (G3) chondrosarcoma (17 samples) and dedifferentiated chondrosarcoma (16 samples). The *SOX9* gene expression level was significantly increased in G1, 2 and 3 sarcoma compared to healthy cartilage, but is not changed significantly in dedifferentiated chondrosarcoma compared to the cartilage control. Additionally, G2 and 3 chondrosarcoma revealed an increased *SOX9* expression compared to dedifferentiated chondrosarcoma (Figure 1A). We confirmed the data from the meta-analysis in part by isolating RNA from three grade II biopsies and comparing the *SOX9* gene expression level with three healthy control cartilage samples (Supplementary Figure S1).

### 2.2. SOX9 Protein Expression in Dedifferentiated Chondrosarcoma

Dedifferentiated chondrosarcoma (DDCS), a highly malignant variant of chondrosarcoma, are characterized by a high-grade sarcoma area next to a low-grade chondroid tumor region. Surprisingly, *SOX9* gene expression is not higher in DDCS compared to normal cartilage, as it was the case for G1, 2 and 3 tumors (Figure 1A). Thus, we analyzed SOX9 protein expression and distribution in DDCS by means of a tissue micro array (TMA) of dedifferentiated chondrosarcoma tissue samples from different patients, with immunohistochemical (IHC) staining of SOX9. The tumor samples were spotted as triplets and represent different areas of the tumor (complete list in Supplementary Figure S2). Thereby, a clear difference was observed between SOX9 reactivity in low-grade (highly differentiated and still cartilaginous) and high-grade, dedifferentiated (non-cartilaginous) parts of the sarcoma samples (Figure 1B). Almost all analyzed chondroid areas of the sarcoma samples were strongly positive for SOX9 (17/19), whereas in the dedifferentiated high-grade parts only three out of 29 samples revealed SOX9 reactivity in single cells (Figure 1C).

### 2.3. Generation of siRNA-Mediated SOX9 Knockdown and Crispr/Cas9-Mediated SOX9 Knockout Clones

**Figure 2:**
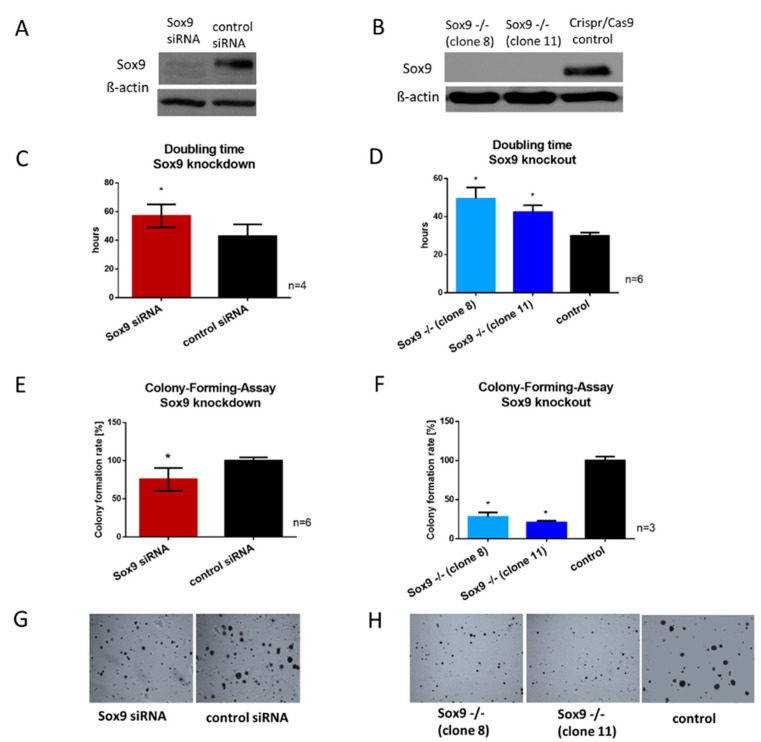
Proliferation and colony-forming ability after Sox9 knockout and knockdown in HTB94 chondrosarcoma cells. (**A**) Representative Western blot images of SOX9 after siRNA-mediated SOX9 knockdown and Crispr/Cas9-mediated SOX9 knockout (**B**). (**C**,**D**) SOX9 knockdown and knockout cells revealed an increase in doubling time and (**E**,**F**) a decrease in the ability to form colonies from single cells cultured in agarose. (**G**,**H**) Representative images of colony formation after SOX9 knockdown and knockout (Magnification 10×). Results are the means ± SD; * *p* ≤ 0.05.

According to the data shown in Figure 1, we assumed an important role for SOX9 in tumor development, progression and differentiation/dedifferentiation status, and we hypothesized that the reduction or depletion of SOX9 potentially influences cancerogenic characteristics in chondrosarcoma. Thus, we transiently reduced SOX9 expression in a chondrosarcoma cell line (HTB94, derived from a G2 chondrosarcoma) via siRNA, leading to a decrease in SOX9 gene expression (Supplementary Figure S3A) and accordingly, to decreased protein expression by 80–90% compared to control cells (non-targeting siRNA = nt) (Figure 2A). In addition to the transient siRNA-based knockdown of SOX9, with the disadvantage of time-limited effects and in order to compare the effects of the total absence of SOX9 with effects of reduced expression, we completely depleted SOX9 via Crispr/Cas9 technology in HTB94 cells. Two single-cell-derived colonies (clones 8 and 11) with complete SOX9 knockout were selected and sequenced (Supplementary Figure S3B) to verify the generated mutations. In the two selected clones, both SOX9 alleles were disrupted by deletion or insertion resulting in a total SOX9 knockout, which was verified via Western blotting (Figure 2B). The SOX9 knockout clones showed slight morphological variations (Supplementary Figure S3C) due to their single cell origin from the typically heterocellular tumor cell line HTB94.

### 2.4. SOX9 Knockdown and Knockout Impair Proliferation and the Ability to Form Colonies

Since SOX9 is associated with proliferation in various other cell types, we examined the effect of SOX9 inhibition on the growth rate of chondrosarcoma cells. The doubling time of both SOX9 knockdown and knockout cells was significantly extended. After siRNA application, SOX9 knockdown cells had a mean doubling time of 57 h and the control cells, transfected with non-targeting siRNA, a mean doubling time of 43 h (Figure 2C). The two SOX9 knockout clones revealed their average doubling times between 42 and 49 h, whereas the control Crispr/Cas9 clone revealed a mean doubling time of 30 h (Figure 2D).

The ability to form adhesion-independent colonies from a single cell is a hallmark in cancer progression and metastasis potential. Therefore, we performed a colony-forming-assay (CFA) in soft agar after SOX9 reduction and depletion. Both SOX9 knockdown and knockout resulted in a diminished ability to form new colonies from single cells, with a 25% reduction for SOX9 knockdown cells (Figure 2E) and on average a 50–75% reduction for SOX9 knockout cells (Figure 2F). Representative images of the colonies after SOX9 reduction or depletion are shown in Figure 2G,H. The results indicated that SOX9 is essential for maintaining proliferation and also the clonogenicity of chondrosarcoma cells.

### 2.5. SOX9 Knockdown and Knockout Increases Apoptosis in Chondrosarcoma Cells Involving BCL-2 and Survivin Expression

**Figure 3:**
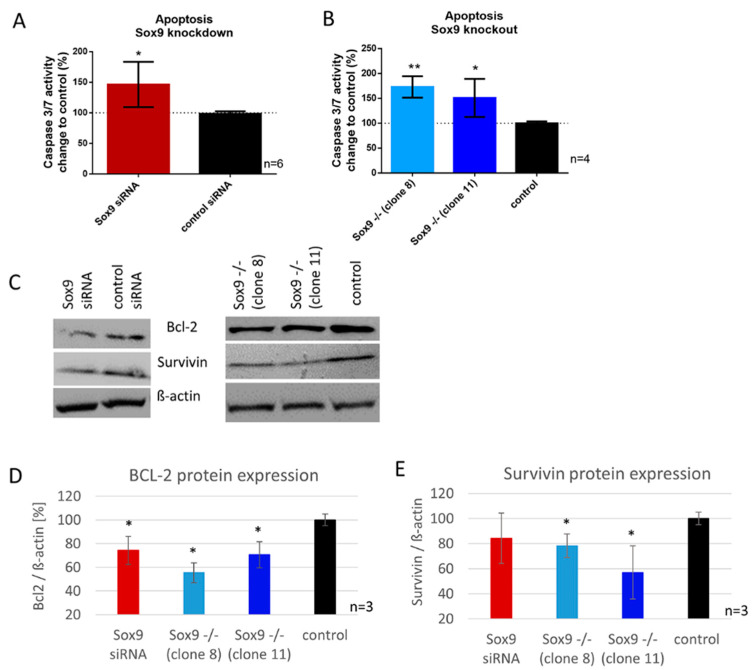
Effect of SOX9 knockdown and knockout on apoptosis. (**A**,**B**) Increased apoptosis in SOX9 knockdown and knockout cells was measured via Caspase 3/7 activity assay. (**C**) Western blot analysis of BCL-2 and survivin after SOX9 knockdown and knockout revealed a decrease in both anti-apoptotic proteins. (**D**,**E**) Densitometric analysis of 3 independent Western blots are shown. * *p* ≤ 0.05, ** *p* ≤ 0.01.

Here, we analyzed the apoptotic rate in SOX9 knockdown, knockout and control cells with a caspase 3/7 activity assay and determined the protein expression of two major apoptosis mediators, BCL-2 and survivin. The reduction of SOX9 (knockdown) resulted in a significant increase in caspase 3/7 activity (48%), correlating with increased apoptosis (Figure 3A). SOX9 knockout clone 8 showed 73% and SOX9 knockout clone 11, 48% increase in caspase 3/7 activity (Figure 3B). Protein synthesis of survivin was significantly reduced in both SOX9 knockout clones, and in SOX9 knockdown cells by trend. Comparable results were observed for the anti-apoptosis protein BCL-2. SOX9 knockout clones 8 and 11, as well as SOX9 knockdown cells, showed a significantly reduced BCL-2 protein amount (Figure 3C–E).

### 2.6. SOX9 Knockout Increases Cell Adhesion and Decreases Cell Migration Accompanied by the Inability to Activate MMP13

**Figure 4:**
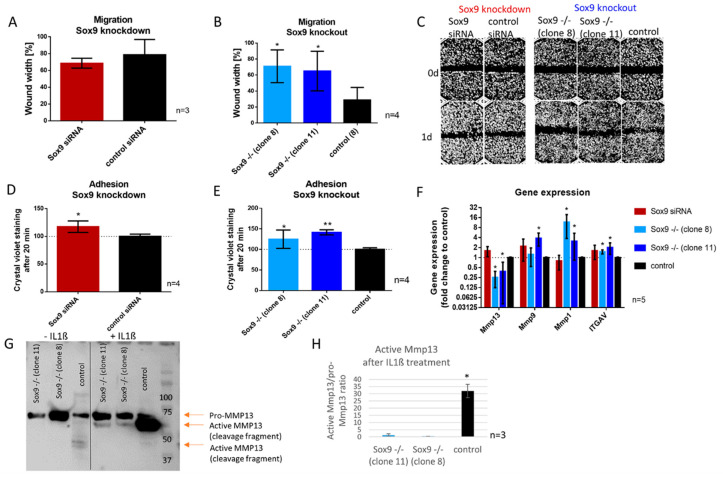
Effect of SOX9 knockdown and knockout on migration and adhesion. (**A**,**B**) Migration (wound healing) assay of SOX9 knockdown and knockout cells showed decreased migration (wider wound) in SOX9 knockout clones 8 and 11 compared to the control. (**C**) Representative pictures of wound healing assays after SOX9 knockdown and knockout (Magnification 4×). (**D**,**E**) Adhesion to cell culture plastic surface increased significantly after SOX9 knockdown and knockout. (**F**) Gene expression analysis showed a decreased expression of *MMP13* and an increased expression of *MMP9* and *MMP1* and *ITGAV* after SOX9 knockout. (**G**) Western blot analysis of MMP13 revealed a hampered ability to activate MMP13 in SOX9 knockout clones after IL-1β stimulation. (**H**) Densitometric analysis of 3 independent Western blots is shown. Results are the means ± SD; * *p* ≤ 0.05, ** *p* ≤ 0.01.

Wound healing assays were performed after SOX9 knockdown and knockout to investigate the migratory ability in those cells. Whereas SOX9 knockdown cells did not show a significant difference in migration compared to control cells (Figure 4A), SOX9 knockout clone 8 and 11 left a significantly wider gap in the cell layer one day after scratch introduction, indicating a reduced migration rate when Sox9 was depleted (Figure 4B). Representative images of the migration assay are shown in Figure 4C. As alterations in migration are potentially related to changes in adhesion capacity, we analyzed the adhesion potential of cells on cell culture dishes 20 min after seeding (Figure 4D,E) and in addition, a panel of migration and adhesion-related genes after SOX9 knockdown and knockout (Figure 4F). Thereby, SOX9 knockdown and knockout cells showed an increased proportion of adherent cells compared to the control (Figure 4D,E). Gene expression analysis after SOX9 knockdown and knockout displayed a significant decrease in *MMP13* and an increase in *MMP1* and *ITGAV* gene expression in both SOX9 knockout clones and an increase in *MMP9* in one of the two clones (Figure 4F). In accordance, the analysis of MMP13 at the protein level, revealed a strongly reduced ability to activate MMP13 after IL-1β treatment in SOX9 knockout cells (Figure 4G,H).

### 2.7. SOX9 Depletion Induces Polyploidy

**Figure 5:**
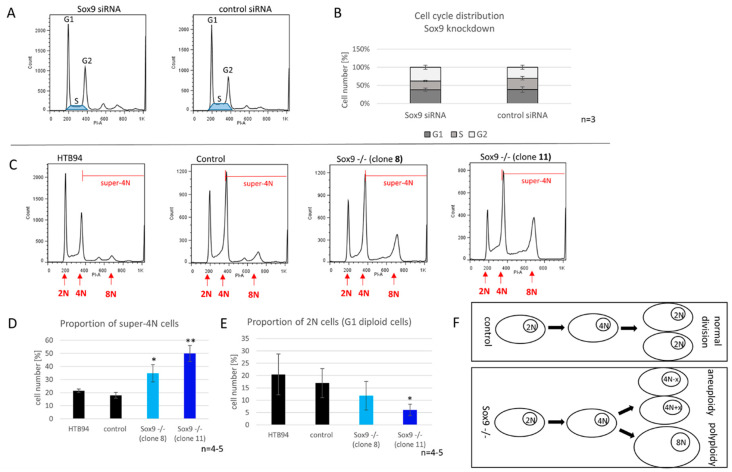
Cell cycle distribution of SOX9 knockdown and knockout cells. (**A**) Representative flowcytometric cell cycle analysis of SOX9 knockdown cells and the distribution of cell cycles phases after SOX9 knockdown (**B**). (**C**) Representative flowcytometric cell cycle analysis of SOX9 knockout clones. The area of super-4N cells (4N = DNA content after replication and before mitosis) is marked with red lines. (**D**) The proportion of super-4N cells is significantly increased in SOX9 knockout clones 8 and 11 compared to control. (**E**) The number of cells in G1-phase was reduced by trend in SOX9 knockout clone 8 and reduced significantly in the SOX9 knockout clone 11 compared to the control. (**F**) The development of aneuploidy or polyploidy after the SOX9 knockout. Control cells do not (or to a much smaller extent) exhibit genetic instability and maintain a diploid genome after division, whereas Sox9^−/−^ cells reveal abnormalities during division leading to aneuploid and/or polyploid cells. Results are the means ± SD; * *p* ≤ 0.05; ** *p* ≤ 0.01.

We evaluated the effect of reduced and depleted SOX9 expression on the cell cycle of synchronized chondrosarcoma cells. After the SOX9 knockdown, we observed a tendency for a reduced proportion of S-phase cells. On average, control cells had an S-phase proportion of 22%, whereas in the SOX9 knockdown group, less than 17% of the cells were in the S-phase (Figure 5A,B).

Cells without SOX9 (knockout) revealed a significantly increased number of aneuploid and polyploid (super-4N) cells. Hyperdiploidy (47, XX, +7; according to ATCC) was already reported in the original cell line HTB94. In line with this, we determined a proportion of ~20% of super-4N cells (cells with more than a 2-fold chromosome set before mitosis) in both control Crispr/Cas9 cells and untreated HTB94. Interestingly, the depletion of SOX9 resulted in a significant increase in super-4N cells in both SOX9 knockout clones (Figure 5C,D) accompanied by a reduced proportion of cells in the G1-phase (Figure 5E). The 8N-peak especially increased remarkably in the cell clones lacking SOX9, indicating an important role of SOX9 for maintaining genetic stability and inhibiting numerical alterations of chromosomes in chondrosarcoma cells (Figure 5F).

### 2.8. SOX9 Depletion Alters Sensitivity against Doxorubicin and against Infection with Oncolytic Virus (T-VEC)

**Figure 6:**
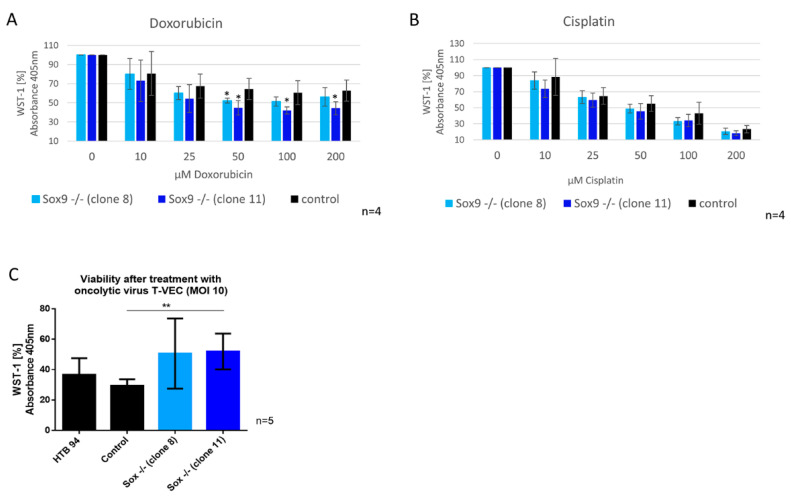
Viability of the SOX9 knockdown and knockout cells after doxorubicin and oncolytic virus treatment. (**A**) Increased sensitivity against doxorubicin after SOX9 knockout (50, 100, 200 µM dx), but not against cisplatin (**B**). (**C**) Decreased sensitivity against oncolytic virus (T-VEC) treatment after SOX9 knockout. Results are the means ± SD; * *p* ≤ 0.05; ** *p* ≤ 0.01; MOI = multiplicity of infection.

We hypothesized that SOX9 knockout cells may show a different sensitivity to therapy approaches due to their difference in functional cancerogenic properties. Therefore, we conducted a WST-1 viability assay to analyze whether SOX9 knockout cells have different sensitivities to doxorubicin and cisplatin. In addition, we performed a WST-1 assay with SOX9 knockout cells after applying a potential new treatment option, infection with an oncolytic virus (T-VEC, approved for melanoma cancer therapy). The viability of the two SOX9 knockout clones decreased significantly after treatment with 50 µM doxorubicin (10–15% reduction) (Figure 6A), whereas cisplatin had no significant effect (Figure 6B). Infection with the oncolytic virus (T-VEC) revealed that SOX9 knockout clone 11 retained significantly more viable cells after T-VEC infection with an MOI (multiplicity of infection) of 10, and SOX9 knockout clone 8 showed the same result by trend (Figure 6C), implying a lower potential efficiency of this anticancer therapy in chondrosarcoma cells without SOX9 expression.

## 3. Discussion

The versatile function of SOX9 has been studied extensively from a developmental point of view, particularly during embryonic chondrogenesis and male gonad genesis [13]. Notably, stem cell homeostasis and the dysregulation of tissue differentiation pathways can critically contribute to the onset and progression of cancer. Experimental and clinical data revealed an important role for SOX9 in tumorigenesis as it is overexpressed in a variety of human cancers like hepatocellular carcinoma, prostate, bladder, gastric, breast, ovarian, pancreatic and colorectal cancer, where its expression correlates with tumor progression and malignancy [14,15]. Surprisingly, the role of SOX9 in chondrosarcoma is still not understood, although SOX9 is the master transcription factor of chondrogenesis. Performing a meta-analysis of intrinsic gene expression levels of *SOX9* in human chondrosarcoma biopsies, revealed an increase in *SOX9* in grade I (G1), II (G2) and III (G3) tumors compared to cartilage, but interestingly no significant difference between dedifferentiated chondrosarcoma (DDCS) and cartilage was found. This decrease in *SOX9* in DDCS in comparison to high-grade chondrosarcoma indicates that SOX9 reduction may support the progression into dedifferentiated stages or could even be a requirement for it. Other in vitro studies recently showed, that tumor progression involved SOX9 and CDKN1C repression in different chondrosarcoma cell lines [12]. Posttranscriptional regulation of SOX9 via miRs, i.e., miR-145, might be involved in this mechanism as reported by Mak et al. [16]. DDCS are known to be a highly malignant variant of chondrosarcoma. They are defined as a neoplasm containing two different components: a low-grade chondroid, well differentiated compartment and a high-grade, malignant and dedifferentiated compartment. Owing to early metastasis and poor response to chemotherapy, DDCS have a poor prognosis with a 5 year survival rate of 10–24% [7]. We showed via TMA that SOX9 is almost exclusively and highly expressed in the low-grade, cartilaginous compartment of DDCS, but not in the dedifferentiated parts, and might thereby be useful as a marker to distinguish between the two different areas within the sarcoma. These findings are in line with the data of Tang et al., indicating that SOX9 is, together with RUNX2, involved in the emergence and development of DDCS [17] and that SOX9 immunostaining is a helpful therapeutic tool to distinguish chondrosarcoma from various small round cell tumors such as small cell osteosarcomas, non-Hodgkin lymphomas and Ewing/PNET (primitive neuroectodermal tumor) family tumors [18]. The rare genetic reports on dedifferentiated chondrosarcoma demonstrate that both components share identical genetic aberrations [19,20], with additional genetic changes in the anaplastic component [19,20,21,22], indicating a common precursor cell with an early diversion of the two components. Chromosomal structural abnormalities and genetic instability are reported in well differentiated chondrosarcoma analyzed by cytogenetics, and it is assumed that a substantial subset of chondrosarcoma start as hyperhaploid or hypodiploid tumors, followed by polyploidization, a hypothesis previously put forward based on cytogenetic data as well as the loss of heterozygosity data [23,24]. One driver of this genetic stability and structural normality is probably SOX9, as the depletion of the transcription factor revealed a massive increase in the proportion of polyploid and aneuploid cells (super-4N) in our experiments. It is known that exclusively cells with a defective intrinsic apoptotic signaling pathway are able to survive such ploidy changes and to pass the mitotic spindle checkpoint [25]. Polyploidy is caused by the failure of the mitotic checkpoint and the endo-replication of chromosomal DNA, and is a prerequisite for aneuploidy and genomic instability leading to the development and progression of human neoplasms [26,27]. Once accomplished, polyploidy may cause tumor progression in various forms, including the development of chromosomal aberrations leading to the duplication or loss of genes and changes in epigenetic chromatin [26,27], but could also lead to the apoptosis of the tumor cells if the genetic abnormalities are too massive [28]. In the early stages of tumor formation, it was assumed that tetraploid cells lead to aneuploid cells. The proliferation of tetraploid cells is limited by the active tetraploidy checkpoint (4N G1), and tetraploid cells that are able to cross this control point lead to enhanced tumor progression and genomic instability [29]. Therefore, we suppose that SOX9 influences the development of karyotypic aberrations and the accumulation of genetic mutations in oncogenesis, thereby probably affecting the way and time of tumor development and progression. The connection between ploidy and prognosis in chondrosarcoma was recognized almost 40 years ago. Kreicbergs et al. analyzed tumors with a highly malignant clinical progression (death occurring within two years) and showed that the primary tumors were hyperploid in 81% of the analyzed cases of metastatic chondrosarcomas, which means that these tumors had increased levels of nuclear DNA [6]. Our data indicate that SOX9 is essential for preventing genetic instability in chondrosarcoma cells and that a threshold of SOX9 expression/activity for this mechanism may exist, as we did not observe this dramatic increase in aneuploid and polyploid cells after SOX9 knockdown. However, both SOX9 knockdown and knockout cells exhibit a strong increase in apoptosis, accompanied by a reduction of the anti-apoptosis mediator BCL-2 suggesting a potential direct connection between SOX9 and BCL-2 during chondrosarcoma progression to a dedifferentiated stage. Similar results were shown by Chen et al. in chordoma cells, a rare type of sarcoma that occurs in the bones of the skull base and spine. They reported that a transient siRNA-based SOX9 knockdown in a chordoma cell line leads to enhanced apoptotic activity [30]. In line with their data, we observed, a reduction of survivin in the SOX9 knockout clones, but not in the SOX9 knockdown cells. We thus suggested that SOX9 is definitely involved in the regulation of apoptosis and may also contribute to drug resistance via this link. As the suppression of apoptosis by the acquisition of mutations is considered to be a hallmark of cancer, we concluded that SOX9 drives the accumulation of mutations, genetic and chromosomal modifications and thereby functions as a critical oncogene in chondrosarcoma cancer progression. 

Previously, we showed that the SOX9 level is crucial for the proliferation, apoptosis and viability of rat mesenchymal stem cells [31]. Our present results demonstrate that the knockdown and knockout of SOX9 in HTB94 cells drastically reduced the proliferation rate and hampered clonogenicity and migratory ability. While an increase in proliferation is important for the initiation and maintenance of primary tumors, growth inhibition, as we observed after the depletion of SOX9, could ultimately be crucial for the survival of carcinoma cells during migrative or invasive steps of carcinogenesis, like the extravasation into secondary organs, thereby leading to the development of a more malignant phenotype [32]. This points to a pro-survival role of SOX9 in chondrosarcoma cells during early cancer stages, promoting tumor development, growth and progression, but at the same time, SOX9 may act as an inhibitor for progression into dedifferentiated tissue. However, it must be kept in mind that these results were obtained with a grade II-derived chondrosarcoma cell line and that the role of SOX9 must be assessed as strictly stage specific. SOX9 seemed to affect either directly or indirectly the expression and activity of different integrins and matrix-metalloproteinases (MMPs) in chondrosarcoma cells, giving evidence to an involvement into metastatic potential. We observed almost no ability to activate MMP13 after IL-1ß treatment when SOX9 was missing, leading to the assumption of hampered metastatic capacity in those cells. The main substrate of MMP13 is type II collagen, the major collagenous component of cartilage. Thus, cleaving type II collagen by MMP13 may favor invasion and motility potential in chondrosarcoma. For gastric cancer, SOX9 was identified as a critical determinant in the control of epithelial-to-mesenchymal transition (EMT) and metastasis via affecting MMP-2 and MMP-9 [33]. For melanoma cells, it was reported that the overexpression of SOX9 can also promote the invasiveness of the parental melanoma cells by modulating the expression of various MMPs [34]. MMP13 was suggested to be associated with advanced local invasion and aggressiveness in oral and head and neck squamous cell carcinoma [35]. In line with this, we claim a direct effect of Sox9 on the aggressiveness of chondrosarcoma via MMP13. However, in contrast, we observed different results for MMP-1 and MMP-9 in the two SOX9 knockout clones. SOX9 knockout clone 8 showed increased *MMP-1* gene expression level, whereas clone 11 displayed increased *MMP-9* gene expression. Therefore, we assumed possible SOX9-independent effects on different MMPs, possibly due to the different additional mutations within the heterogenic population of the chondrosarcoma cell line.

The role of SOX9 in chemoresistance has been reported for multiple tumors as for breast cancer, glioblastoma, cervical cancer, gastric cancer and lung cancer [36,37,38,39,40,41,42]. Yuan et al. showed for intrahepatic cholangiocarcinoma cells [36], that SOX9 knockdown or knockout did not impact the efficiency of Cisplatin. We observed the same for chondrosarcoma cells. However, both SOX9 knockout clones enhanced the cytotoxic effect of doxorubicin in chondrosarcoma cells. This was most obvious at a concentration of 50 µM, indicating that the potential of SOX9 to change the sensitivity for this cancer drug, depending on its expression levels, could be useful in a personalized treatment strategy. A novel field in cancer treatment research, the use of the oncolytic virus, provides hope to potentially overcome chemoresistance in chondrosarcoma. Here, we provide results for the first time showing that the loss of SOX9 in chondrosarcoma cells increases the resistance against the oncolytic virus therapy using T-VEC (Talimogen laherparepvec). T-VEC is a type I herpes simplex virus genetically modified to preferentially replicate in tumor cells, enhance the antigen loading of MHC class I molecules and express granulocyte–macrophage colony-stimulating factor (GM-CSF) to increase tumor-antigen presentation by dendritic cells. The T-VEC virus has a built-in safety mechanism through the thymidine kinase (TK) gene. A deletion of the TK gene causes the attenuation of the virus in non-dividing cells. That property may give it some selectivity for replication in cycling cancer cells [43]. It is currently the only oncolytic virus approved by the FDA with an indication for advanced melanoma based on an improved sustained response rate in a randomized, phase III trial [44]. As we observed a clear difference in the proliferative behavior in the SOX9 knockout clones compared to the control chondrosarcoma cells, we assumed a potentially different sensitivity to oncolytic virus treatment. The two opposing treatment effects of doxorubicin and oncolytic virus which we observed in our study approach in SOX9 knockout cells might result from different underlying mechanisms. Doxorubicin blocks the proliferation of cancer cells through proteolytic activation of CREB3L1 [45], whereas T-VEC kills tumor cells via lytic replication [46]. Usually, the susceptibility of tumors towards oncolytic viruses increases with their metastatic potential, as shown for malignant melanoma, which was associated with cumulative defects in STING-cGAS signaling [47]. However, the targeted knockout of SOX9 in HTB94 cells may point to a different role of this molecule in oncolytic herpes virus infection: SOX9 may be required to ascertain sufficient cell survival to allow lytic virus replication. To date, the influence of SOX9 on this mechanism has not been studied in detail. Especially in terms of T-VEC treatment, it is necessary to understand the biological processes underlying the immune dysregulation in the chondrosarcoma cells lacking SOX9 to understand the limitation of this therapy option.

## 4. Material and Methods

### 4.1. Meta-Analysis of SOX9 Gene Expression Level

For the meta-analysis of the SOX9 gene expression level, the TCGA, GEO and Array Express databases were used. The clinical values of Sox9 expression in chondrosarcoma was investigated by collecting and calculating mRNA-Seq data from the TCGA database, and the statistical analysis was performed in R 3.5.0. GEO and Array Express databases were used for the normal cartilage data organization of 26 cartilage samples. (GSE51588 (https://www.ncbi.nlm.nih.gov/geo/query/acc.cgi?acc=GSE51588), GSE1919 (https://www.ncbi.nlm.nih.gov/geo/query/acc.cgi?acc=GSE1919), GSE117999 (https://www.ncbi.nlm.nih.gov/geo/query/acc.cgi?acc=GSE117999), E-MTAB-5564 (https://www.ebi.ac.uk/arrayexpress/experiments/E-MTAB-5564/). Array Express database was used for chondrosarcoma data organization: the raw data of 102 cartilage tumors was downloaded from Array Express (https://www.ebi.ac.uk/arrayexpress/experiments/E-MTAB-7264/).

### 4.2. Cell Culture

HTB94 (SW1353) chondrosarcoma cells were obtained from ATCC and cultured in DMEM-F12 (Merck, Darmstadt, Germany) containing 10% FBS (Gibco/Thermo Fisher, Waltham, MA, USA) and 1% penicillin/streptomycin (Gibco/Thermo Fisher, Waltham, MA, USA) under 5% CO_2_ at 37 °C in a humidified atmosphere.

### 4.3. Immunohistochemistry

The TMA slides were deparaffinized at 60 °C for 1 h and rehydrated in a descending alcohol row. For antigen retrieval, the TMA slides were incubated in citrate buffer (pH 6.0) for 20–24 h at 60 °C in a water bath. After blocking with 5% goat serum (Merck, Darmstadt, Germany) for 1 h (room temperature), incubation with the SOX9 antibody (Cell Signaling Technology (CST), Danvers, MA, USA; 1:600; #826308) occurred (4 °C, overnight). Activation and visualization were performed by using SignalStain^®^ Boost IHC Detection Reagent (HRP (horseradish-peroxidase), rabbit; CST, for 30 min and development was carried out with DAB Substrat (Agilent DAKO, Santa Clara, CA, USA) for 15 min, followed by Gill’s Hematoxylin nuclear staining.

### 4.4. TMA Generation

For the generation of the tissue micro array (TMA), we used tumor samples that were formalin-fixed and paraffine-embedded from the archive of the institute of pathology of the Technical University of Munich (TUM) and assembled the samples using a Tissue Microarrayer (Beecher Instruments, Sun Prairie, WI, USA) with a core size of 0.6 mm. At least 3 or if possible up to 6 tumor nuclei were taken from areas with vital tumor tissue previously marked by a board-certified pathologist.

### 4.5. SOX9 siRNA Transfection

A pool of 5 different SOX9 siRNAs was purchased from Dharmacon (Horizon Discovery Dharmacon, Lafayette, CO, USA) and transfection was performed according to the manufacturer’s instructions at a final concentration of 25 nM for the SOX9 siRNA. A pool of 4 different non-targeting siRNA was used as the negative control. DharmaFECT siRNA Transfection Reagent (Horizon Discovery Dharmacon, Lafayette, CO, USA) was used as the transfection reagent. From 24 to 48 h after transfection, the cells were harvested for RNA or protein extraction or trypsinized and re-seeded for functional assays.

### 4.6. Generation of SOX9−/− Knockout Cells

Crispr/Cas9 SOX9 plasmid (sc-400143-NIC) was purchased from Santa Cruz Biotechnologies (Santa Cruz Biotechnologie, Dallas, TX, USA). The sequences of gRNA are CAGGAGAACACGTTCCCCAA for strand A and CGTGTTCTCGGTGTCCGAGC for strand B. Non-targeting guideRNA (gRNA) was used as the negative control (SC-437281). UltraCruz Transfection Reagent (Santa Cruz Biotechnologie, USA; sc-395739) was used for transfection. According to the manufacturer’s instruction, transfection was carried out in a 6-well plate with a seeding density of 200,000 HTB94 cells/well. A mixture of 2 µg/well DNA and 10 µL UltraCruz Transfection Reagent (Santa Cruz Biotechnologie, USA) was added and incubated for 24 h. Afterwards, this transfection medium was exchanged to a normal medium for another 24 h, followed by selection with 2 µg/mL puromycin (Merck, Darmstadt, Germany) for 4 days. Single cell dilution was carried out via seeding the cells in a 96-well plates at 1 cell per well in standard cell culture media. Single cell-derived colonies were picked and propagated. When the clones reached confluence, cells were trypsinized and an aliquot was kept for protein screening by Western blotting. The remaining cells were frozen or expanded for additional analyses. To identify the status of genome editing, the PCR amplification of the SOX9 gene was performed with genomic DNA isolated with the High Pure PCR Template Preparation Kit (Roche, Munich, Germany #11796828001) from different clonal SOX9 knockout cell lines. In short, we used PCR to amplify the target mutated region, ligated the PCR products into a Topo-TA-cloning plasmid (Invitrogen/Thermo Fisher, Waltham, MA, USA #45-0031), and transformed this plasmid into chemically competent bacteria. After this bacterial subcloning, the mutated SOX9 gene was sequenced and aligned with the wild-type sequence using BLAST (version 4).

### 4.7. Protein Extraction and Western Blot Analysis

For protein extraction, HTB94 cells were washed with PBS, detached with Trypsin–EDTA (Merck, Darmstadt, Germany) and after washing again with PBS, harvested and lysed. Total cell lysates were prepared with RIPA buffer (Invitrogen/Thermo Fisher, Waltham, MA, USA) containing proteinase inhibitor (Roche, Munich, Germany). Protein concentration was quantified with the BCA assay (Invitrogen/Thermo Fisher, Waltham, MA, USA) and lysate aliquots containing 25–50 µg of total protein (depending on the protein of interest) were boiled for 5 min with SDS-sample buffer containing β-mercaptoethanol (Merck, Darmstadt, Germany) and subjected to a 10–15% SDS-PAGE. After electrophoretic separation, the proteins were transferred to nitrocellulose membranes (Bio-Rad, Hercules, CA, USA) and blocked with 5% dried milk (Carl Roth, Karlsruhe, Germany) and subsequently incubated with the following primary antibody for 16 h at 4 °C or 1 h at room temperature: rabbit polyclonal anti-SOX9 (#AB5545, 1:2000; Merck, Darmstadt, Germany), rabbit polyclonal anti-Bcl-2 (#2872, 1:1000; Cell Signaling Technology (CST), Danvers, MA, USA), rabbit polyclonal anti-Survivin (#2808, 1:1000; Cell Signaling Technology (CST), Danvers, MA, USA). Loading was controlled with rabbit monoclonal antibody to β-actin (#ab8227, 1:15.000; Merck, Darmstadt, Germany). After washing, the membranes were incubated with mouse or rabbit HRP (horseradish peroxidase)-coupled secondary antibody (1:10,000, Jackson Immuno Research, West Grove, PA, USA). Proteins were detected using ECL detection reagents (Invitrogen/Thermo Fisher, Waltham, MA, USA). Western blot signals were analyzed densitometrically using Photoshop CS3 and normalized to β-actin [26].

### 4.8. Flow Cytometry

500,000 HTB94 cells per T-175 flask were seeded in complete medium. After 24 h, the cells were washed twice with PBS and cultured in medium without FCS for an additional 24 h to obtain cell synchronization in G0/G1. By adding FCS-containing complete medium again, the cells started to enter the cell cycle and samples were taken 24 h after FCS addition. Cell pellets were washed with cold PBS containing 2% BSA and fixed carefully with a methanol–acetone mixture. Staining of DNA with propidium iodide (Merck, Darmstadt, Germany; 25 µg/mL per 1 million cells in 500 µL) was performed after RNase (Invitrogen/Thermo Fisher, Waltham, MA, USA) treatment for 1 h at 37 °C. Nuclei were analyzed in FACS Canto (Becton Dickinson, Franklin Lakes, NJ, USA) and evaluated with FlowJo [26].

### 4.9. RNA Isolation from Human Tissue Samples (Chondrosarcoma Biopsies)

The collected biopsies (30–80 mg) were directly transferred to RNA-Lysis Buffer (RLT-Buffer, Qiagen, Venlo, The Netherlands and isolated according to the manufacturer’s instruction with the RNeasy Kit (Qiagen, Venlo, The Netherlands).

### 4.10. RNA Isolation and Real-Time RT-PCR from HTB94 Cells

Total cellular RNA was isolated using the Absolutely RNA Miniprep Kit (Stratagene, San Diego, CA, USA) according to the manufacturer’s instructions. To generate single-stranded cDNA, RNA was reverse transcribed with an AffinityScript QPCR cDNA Synthesis Kit (Stratagene, San Diego, CA, USA) and PCR was performed with the Mx3005P QPCR System from Agilent Technologies using Brilliant II SYBER Green qPCR Mastermix (Agilent Technologies, Santa Clara, CA, USA) [26]. Gene expression in HTB94 cells were analyzed relatively, calibrated to the expression in control cells, and normalized to GAPDH, TBP and 18s using the primers shown in Table 1.

### 4.11. Doubling Time

For assaying the cell growth, equal numbers of cells were seeded in 6-well plates. The cells of one well were detached every day and counted with a cell counter (Cedex, Roche, Germany) for up to 6 days. The doubling time was calculated using the following formula:

(1) $$\mathrm{Doubling}\;\mathrm{Time}\;=\;\frac{\mathrm{duration}\;\times \;\log\left(2\right)\;}{\log\left(\mathrm{Final}\;\mathrm{Concentration}\right)-\log\left(\mathrm{Initial}\;\mathrm{Concentration}\right)}$$

### 4.12. Caspase-3/7 Assay

Caspase-3/7 enzymatic activity was measured as an indicator of apoptosis using the Apo-ONE Homogeneous Caspase-3/7 assay (Promega, Fitchburg, WI, USA) according to the manufacturer’s instructions. A non-fluorescent caspase substrate (Z-DEVD-R110), added to the HTB94 cells, was thereby cleaved into fluorescent molecules with an emission maximum at 521 nm [26].

### 4.13. Wound Healing

Wound healing (migration) assays were performed by seeding cells into the Culture-Insert 2 Well (IBIDI, Gräfelfing, Germany). After 24 h, a cell-free gap was created by the removal of the insert and cell migration can be visualized using a bright field microscope. Cells were washed with PBS and cultured from then on in the serum-free medium. Photos were taken at the beginning and after 24 h, and the closure of the gap was determined via Photoshop CS (Adobe Inc., San Jose, CA, USA) and ImageJ.

### 4.14. Colony-Forming Assay

For the soft-agar-colony-forming assay, a bottom layer of agar with complete media was poured and solidified first, followed by an upper layer containing a specified number of cells suspended in medium agar mixture. After 21 days (media were changed twice a week), the number of colonies was counted and the size of the colonies was measured via Photoshop CS, after crystal violet staining.

### 4.15. WST-Assay

Cells were seeded in 96-well culture plates at a density of 10,000 cells/well and were allowed to grow for 1 days. Subsequently, the cells were treated with doxorubicin and cisplatin in different concentrations for 24 h, before the addition of WST-1 solution (Sigma-Aldrich, St. Louis, MO, USA). Absorbance was measured after 30 min, 1 and 2 h at 405 nm. WST-1 is a tetrazolium salt that is particularly useful for cell proliferation and viability assays. Tetrazolium salts are cleaved to formazan by the succinate-tetrazolium reductase system, belonging to the respiratory chain of the mitochondria, and is only active in metabolically intact cells. Thus, measuring the amount of formazan permits forming a conclusion about the viability of the analyzed cells after experimental treatments.

### 4.16. Oncolytic Virus (T-VEC) Infection

T-VEC (Imlygic^®^) was obtained from Amgen (Thousand Oaks, CA, USA). It was propagated in DMEM (Gibco/Thermo Fisher, Waltham, MA, USA) supplemented with 10% heat-inactivated fetal calf serum, 90 U/mL streptomycin, 0.3 mg/mL glutamine, and 200 U/mL penicillin (all PAN Biotech, Aidenbach, Germany). Vero cells infected at 90% confluency were harvested at the peak of virus replication. The cell pellet harvested and subjected to three freeze–thaw cycles. Thereafter, supernatants were filtered through 0.45 µm pore filters, and stored in aliquots at −80 °C. The 50% tissue culture infectious dose (TCID_50_) was determined. The cell viability of SOX9 knockout cell lines was determined using the TACS^®^ MTT Cell Proliferation Assay (R&D Systems, Minneapolis, MN, USA). A total of 10,000 cells/well and condition were plated in 96-well plates and infected the next day. Mock-infected cells served as controls. Two days later, 10 µL of 3,(4,5-dimethylthiazol-2-yl)-2,5-diphenyl-tetrazolium bromide (MTT) reagent was added per well and incubated until the dye was reduced to purple formazan crystals. After the addition of detergent (100 µL/well), the plate was incubated overnight and analyzed at 595 nm using a Bio-Rad microplate reader (Bio-Rad, Hercules, CA, USA).

### 4.17. Statistics

Statistical analysis was performed using Prism 6 (GraphPad Software Inc., San Diego, CA, USA). Results are presented as the means ± SD. Each assay was performed in replicates and repeated at least in 3 independent experiments. Two-tailed Mann–Whitney tests were used as standard nonparametric tests, determining if the medians of the two groups (experimental group vs. control) were significantly different. Exact *p*-values were calculated. A value of *p* ≤ 0.05 was considered statistically significant. For the semi-quantitative analysis of Western blot intensity (or if specifically denoted), unpaired *t*-tests were used.

### 4.18. Ethical Approval

Anonymized human tissue samples for qPCR and TMA were obtained from the orthopedic surgery in the Asklepios Hospital (Regensburg) and at Barmherzige Brueder Hospital (Regensburg) with ethical approval (February 2018) for their use (18-887-101; Ethikkommission (ethics committee), Universität Regensburg, email: ethikkommission@klinik.ukr.de) and at the TUM in Munich (147/17 S) and with patients’ written informed consent. All experiments were performed in accordance with the relevant guidelines and regulations.

## 5. Conclusions

In summary, our data indicate that SOX9 is highly expressed in chondrosarcoma, while decreasing or losing SOX9 in a late-stage sarcoma may be a prerequisite for tumor progression into a dedifferentiated state. SOX9 knockout results in reduced proliferation, clonogenicity and migration, whereas adhesion, apoptosis and genetic instability were favored, involving cell survival mediators like BCL-2 and survivin. Therefore, we suggest that SOX9 functions as a pro-survival factor in HTB94 cells. SOX9 may also act as a putative proto-oncogene, influencing tumor malignancy and aggressiveness, possibly via the activation of MMP13. We claimed that a certain SOX9 threshold was required for avoiding genetic instability (polyploidy) and thus tumor development and progression, and that drug resistance and the sensitivity against the oncolytic virus was influenced by the SOX9 expression level in chondrosarcoma. Our observations provide novel insight into the role of SOX9 in chondrosarcoma and provide a new rationale for analyzing and targeting SOX9 as a novel component in the treatment strategy of chondrosarcoma.

## Figures and Tables

**Table 1:** Primer sequences.

Gen	Forward	Reverse
ITGAV	GGCTGCATATTTCGGATTTTCTG	CCATTCAGCTTTGTCGTCTGG
MMP13	GACTGGTAATGGCATCAAGGGA	CACCGGCAAAAGCCACTTTA
MMP1	GCCAGATTTGCCAAGAGCAG	GAGTTGTCCCGATGATCTCCC
MMP9	GTACCACGGCCAACTACGAC	GCCTTGGAAGATGAATGGAA
SOX9	GTACCCGCACTTGCACAAC	TCTCGCTCTCGTTCAGAAGTC
TBP	GAACATCATGGATCAGAACAACA	ATAGGGATTCCGGGAGTCAT
18s	CTGGATACCGCAGCTAGGAA	GAATTTCACCTCTAGCGGCG

## Supplementary Materials

The following are available online at https://www.mdpi.com/1422-0067/21/20/7627/s1.

## References

[1] Evola F.R., Costarella L., Pavone V., Caff G., Cannavo L., Sessa A., Avondo S., Sessa G. (2017). Biomarkers of Osteosarcoma, Chondrosarcoma, and Ewing Sarcoma. Front. Pharmacol..

[2] Wesolowski R., Budd G.T. (2010). Use of chemotherapy for patients with bone and soft-tissue sarcomas. Clevel. Clin. J. Med..

[3] Onishi A.C., Hincker A.M., Lee F.Y. (2011). Surmounting chemotherapy and radioresistance in chondrosarcoma: Molecular mechanisms and therapeutic targets. Sarcoma.

[4] Gelderblom H., Hogendoorn P.C., Dijkstra S.D., van Rijswijk C.S., Krol A.D., Taminiau A.H., Bovee J.V. (2008). The clinical approach towards chondrosarcoma. Oncology.

[5] Evans H.L., Ayala A.G., Romsdahl M.M. (1977). Prognostic factors in chondrosarcoma of bone: A clinicopathologic analysis with emphasis on histologic grading. Cancer.

[6] Kreicbergs A., Zetterberg A., Soderberg G. (1980). The prognostic significance of nuclear DNA content in chondrosarcoma. Anal. Quant. Cytol..

[7] Grimer R.J., Gosheger G., Taminiau A., Biau D., Matejovsky Z., Kollender Y., San-Julian M., Gherlinzoni F., Ferrari C. (2007). Dedifferentiated chondrosarcoma: Prognostic factors and outcome from a European group. Eur. J. Cancer.

[8] Mercuri M., Campanacci L. (1995). Dedifferentiated chondrosarcoma. Skelet. Radiol..

[9] Bi W., Deng J.M., Zhang Z., Behringer R.R., de Crombrugghe B. (1999). Sox9 is required for cartilage formation. Nat. Genet..

[10] Wang H.Y., Lian P., Zheng P.S. (2015). SOX9, a potential tumor suppressor in cervical cancer, transactivates p21WAF1/CIP1 and suppresses cervical tumor growth. Oncotarget.

[11] Prevostel C., Rammah-Bouazza C., Trauchessec H., Canterel-Thouennon L., Busson M., Ychou M., Blache P. (2016). SOX9 is an atypical intestinal tumor suppressor controlling the oncogenic Wnt/ss-catenin signaling. Oncotarget.

[12] Nakagawa M., Nakatani F., Matsunaga H., Seki T., Endo M., Ogawara Y., Machida Y., Katsumoto T., Yamagata K., Hattori A. (2019). Selective inhibition of mutant IDH1 by DS-1001b ameliorates aberrant histone modifications and impairs tumor activity in chondrosarcoma. Oncogene.

[13] Jo A., Denduluri S., Zhang B., Wang Z., Yin L., Yan Z., Kang R., Shi L.L., Mok J., Lee M.J. (2014). The versatile functions of Sox9 in development, stem cells, and human diseases. Genes Dis..

[14] Matheu A., Collado M., Wise C., Manterola L., Cekaite L., Tye A.J., Canamero M., Bujanda L., Schedl A., Cheah K.S. (2012). Oncogenicity of the developmental transcription factor Sox9. Cancer Res..

[15] Aguilar-Medina M., Avendano-Felix M., Lizarraga-Verdugo E., Bermudez M., Romero-Quintana J.G., Ramos-Payan R., Ruiz-Garcia E., Lopez-Camarillo C. (2019). SOX9 Stem-Cell Factor: Clinical and Functional Relevance in Cancer. J. Oncol..

[16] Mak I.W., Singh S., Turcotte R., Ghert M. (2015). The epigenetic regulation of SOX9 by miR-145 in human chondrosarcoma. J. Cell. Biochem..

[17] Tang X., Lu X., Guo W., Ren T., Zhao H., Zhao F., Tang G. (2010). Different expression of Sox9 and Runx2 between chondrosarcoma and dedifferentiated chondrosarcoma cell line. Eur. J. Cancer Prev. Off. J. Eur. Cancer Prev. Organ..

[18] Wehrli B.M., Huang W., De Crombrugghe B., Ayala A.G., Czerniak B. (2003). Sox9, a master regulator of chondrogenesis, distinguishes mesenchymal chondrosarcoma from other small blue round cell tumors. Hum. Pathol..

[19] Bovee J.V.M.G., Cleton-Jansen A.M., Rosenberg C., Taminiau A.H.M., Cornelisse C.J., Hogendoorn P.C.W. (1999). Molecular genetic characterization of both components of a dedifferentiated chondrosarcoma, with implications for its histogenesis. J Pathol.

[20] Ropke M., Boltze C., Neumann H.W., Roessner A., Schneider-Stock R. (2003). Genetic and epigenetic alterations in tumor progression in a dedifferentiated chondrosarcoma. Pathol Res. Pract..

[21] Grote H.J., Schneider-Stock R., Neumann W., Roessner A. (2000). Mutation of p53 with loss of heterozygosity in the osteosarcomatous component of a dedifferentiated chondrosarcoma. Virchows Arch. Int. J. Pathol..

[22] Coughlan B., Feliz A., Ishida T., Czerniak B., Dorfman H.D. (1995). p53 expression and DNA ploidy of cartilage lesions. Hum. Pathol..

[23] Bovee J.V., Cleton-Jansen A.M., Kuipers-Dijkshoorn N.J., van den Broek L.J., Taminiau A.H., Cornelisse C.J., Hogendoorn P.C. (1999). Loss of heterozygosity and DNA ploidy point to a diverging genetic mechanism in the origin of peripheral and central chondrosarcoma. Genes Chromosomes Cancer.

[24] Papachristou D.J., Goodman M.A., Cieply K., Hunt J.L., Rao U.N. (2006). Comparison of allelic losses in chondroblastoma and primary chondrosarcoma of bone and correlation with fluorescence in situ hybridization analysis. Hum. Pathol..

[25] Minn A.J., Boise L.H., Thompson C.B. (1996). Expression of Bcl-xL and loss of p53 can cooperate to overcome a cell cycle checkpoint induced by mitotic spindle damage. Genes Dev..

[26] Cleveland D.W., Mao Y., Sullivan K.F. (2003). Centromeres and kinetochores: From epigenetics to mitotic checkpoint signaling. Cell.

[27] Storchova Z., Pellman D. (2004). From polyploidy to aneuploidy, genome instability and cancer. Nat. Rev. Mol. Cell Biol..

[28] Weaver B.A., Cleveland D.W. (2007). Aneuploidy: Instigator and inhibitor of tumorigenesis. Cancer Res..

[29] Mosieniak G., Sikora E. (2010). Polyploidy: The link between senescence and cancer. Curr. Pharm. Des..

[30] Chen H., Garbutt C.C., Spentzos D., Choy E., Hornicek F.J., Duan Z. (2017). Expression and Therapeutic Potential of SOX9 in Chordoma. Clin. Cancer Res. Off. J. Am. Assoc. Cancer Res..

[31] Stockl S., Bauer R.J., Bosserhoff A.K., Gottl C., Grifka J., Grassel S. (2013). Sox9 modulates cell survival and adipogenic differentiation of multipotent adult rat mesenchymal stem cells. J. Cell Sci..

[32] Evdokimova V., Tognon C., Ng T., Sorensen P.H. (2009). Reduced proliferation and enhanced migration: Two sides of the same coin? Molecular mechanisms of metastatic progression by YB-1. Cell Cycle.

[33] Li T., Huang H., Shi G., Zhao L., Li T., Zhang Z., Liu R., Hu Y., Liu H., Yu J. (2018). TGF-beta1-SOX9 axis-inducible COL10A1 promotes invasion and metastasis in gastric cancer via epithelial-to-mesenchymal transition. Cell Death Dis..

[34] Yang X., Liang R., Liu C., Liu J.A., Cheung M.P.L., Liu X., Man O.Y., Guan X.Y., Lung H.L., Cheung M. (2019). SOX9 is a dose-dependent metastatic fate determinant in melanoma. J. Exp. Clin. Cancer Res. CR.

[35] Johansson N., Airola K., Grenman R., Kariniemi A.L., Saarialho-Kere U., Kahari V.M. (1997). Expression of collagenase-3 (matrix metalloproteinase-13) in squamous cell carcinomas of the head and neck. Am. J. Pathol..

[36] Yuan X., Li J., Coulouarn C., Lin T., Sulpice L., Bergeat D., De La Torre C., Liebe R., Gretz N., Ebert M.P.A. (2018). SOX9 expression decreases survival of patients with intrahepatic cholangiocarcinoma by conferring chemoresistance. Br. J. Cancer.

[37] Chen W., Zhao W., Zhang L., Wang L., Wang J., Wan Z., Hong Y., Yu L. (2017). MALAT1-miR-101-SOX9 feedback loop modulates the chemo-resistance of lung cancer cell to DDP via Wnt signaling pathway. Oncotarget.

[38] Feng C., Ma F., Hu C., Ma J.A., Wang J., Zhang Y., Wu F., Hou T., Jiang S., Wang Y. (2018). SOX9/miR-130a/CTR1 axis modulates DDP-resistance of cervical cancer cell. Cell Cycle.

[39] Garros-Regulez L., Aldaz P., Arrizabalaga O., Moncho-Amor V., Carrasco-Garcia E., Manterola L., Moreno-Cugnon L., Barrena C., Villanua J., Ruiz I. (2016). mTOR inhibition decreases SOX2-SOX9 mediated glioma stem cell activity and temozolomide resistance. Expert Opin. Ther. Targets.

[40] Liang Z., Bian X., Shim H. (2016). Downregulation of microRNA-206 promotes invasion and angiogenesis of triple negative breast cancer. Biochem. Biophys. Res. Commun..

[41] Riemenschnitter C., Teleki I., Tischler V., Guo W., Varga Z. (2013). Stability and prognostic value of Slug, Sox9 and Sox10 expression in breast cancers treated with neoadjuvant chemotherapy. SpringerPlus.

[42] Wang J., Xue X., Hong H., Qin M., Zhou J., Sun Q., Liang H., Gao L. (2017). Upregulation of microRNA-524-5p enhances the cisplatin sensitivity of gastric cancer cells by modulating proliferation and metastasis via targeting SOX9. Oncotarget.

[43] Hawkins L.K., Lemoine N.R., Kirn D. (2002). Oncolytic biotherapy: A novel therapeutic plafform. Lancet. Oncol..

[44] Conry R.M., Westbrook B., McKee S., Norwood T.G. (2018). Talimogene laherparepvec: First in class oncolytic virotherapy. Hum. Vaccines Immunother..

[45] Denard B., Lee C., Ye J. (2012). Doxorubicin blocks proliferation of cancer cells through proteolytic activation of CREB3L1. eLife.

[46] Bommareddy P.K., Shettigar M., Kaufman H.L. (2018). Integrating oncolytic viruses in combination cancer immunotherapy. Nat. Rev. Immunol..

[47] Xia T., Konno H., Barber G.N. (2016). Recurrent Loss of STING Signaling in Melanoma Correlates with Susceptibility to Viral Oncolysis. Cancer Res..

