# Supplementary material for: SOX9 Knockout Induces Polyploidy and Changes Sensitivity to Tumor Treatment Strategies in a Chondrosarcoma Cell Line
Source: Int J Mol Sci. 2020 Oct 15;21(20):7627. doi: 10.3390/ijms21207627 (PMC7589851; doi:10.3390/ijms21207627)
Supplement: Supplementary file 1 [file ijms-21-07627-s001.pdf]

Supplementary Materials

SOX9 Knockout Induces Polyploidy and Changes Sensitivity to Tumor Treatment Strategies in a Chondrosarcoma Cell Line

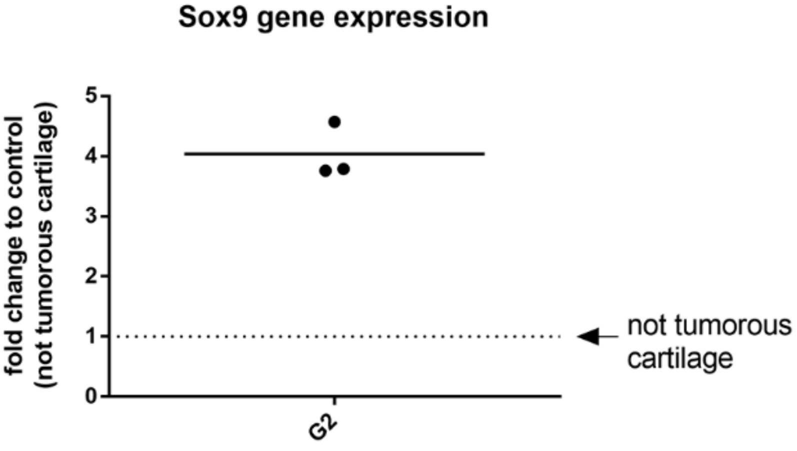

Figure S1. *SOX9* gene expression in 3 biopsies from grade II (G2) chondrosarcoma were analysed and compared to 3 not tumorous cartilage tissue samples.

Sample no	dedifferentiated area	highly-differentiated area
663/15	negative	N/A
11466/14 B	negative	N/A
5869/14 IVB	negative	N/A
5869/14 IVA	negative	positive
11115/13F	negative	N/A
11115/13 G	N/A	positive
11115/13A	negative	positive (G2)
3744/13K	negative	N/A
7240/13 G	negative	positive (weak, G3)
1977/12 IIAT	N/A	N/A
1977/12 IIAN	negative	N/A
1977/12 IIAO	negative	positive
24422/11 ICH	negative	N/A
16770/11 Z7	negative	N/A
16770/11A	N/A	N/A
16770/11 D	negative	N/A
19840/10 IIF	negative	positive
16295/09 IA	N/A	positive (G2-G3)
8314/09 IC	negative	positive (G2-G3)
17775/08 IIB	negative	N/A
17775/08 IIA	N/A	positive (G2)
14420/05 IIIE	weakly positive (single cells)	positive
9646/05 IA	negative	N/A
11362/04 IA	weakly positive (single cells)	N/A
11273/04 Z6	N/A	positive (G3)
11273/04 Z3	N/A	positive (G3)
6266/03 B	negative	N/A
6266/03 F	negative	positive
17359/02 B	negative	negative
17359//02 F	weakly positive (single cells)	N/A
19255/00 IID	N/A	positive (G2-G3)
8108/00	positive	positive
4998/00	N/A	positive (G3)
8580/12 A	N/A	N/A
4681/04 H	positive	N/A
21764/04 D, C	weakly positive (single cells)	positive
21191/04 I C H	N/A	N/A
20410/05 I V, IS	weakly positive (single cells)	positive
11907/07 IA	positive	N/A
3319/15	N/A	N/A
11211/15 Z3	negative	N/A

Figure S2. Summary of the TMA analysis. Dedifferentiated and highly differentiated areas of the chondrosarcoma tissue were evaluated for SOX9 reactivity. Each tumor sample was spotted as triplet on the TMA.

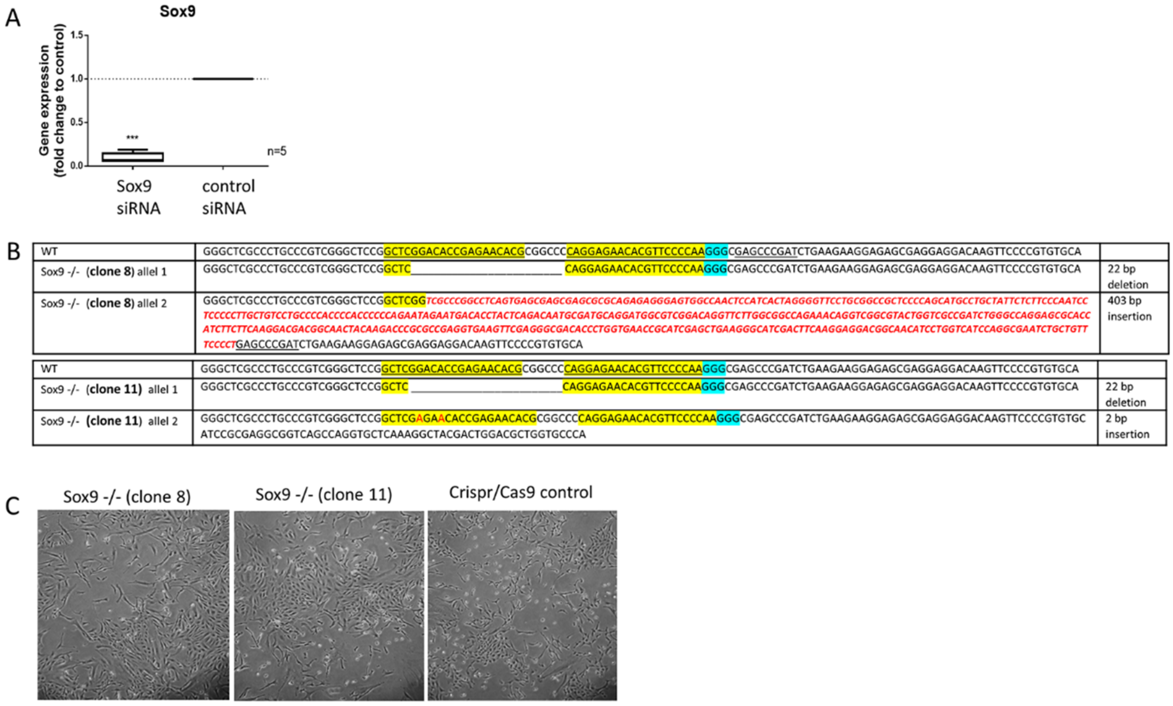

Figure S3. Generation of SOX9 knock<u>down</u> and knock<u>out</u> clones in HTB94 cells. (**A**) SOX9 knockdown after siRNA transfection leads to a 80–90% reduction of gene expression. (**B**) Alignment of the DNA sequences of SOX9 knockout clones with the wild type (WT) sequence. Guide RNA (gRNA) targeting sites are highlighted in yellow, PAM sequence in light blue and inserted nucleotides in red. Deleted parts are marked as low line. (**C**) Morphological appearance of the SOX9 knockout clones which are of single-cell derived origin (magnification 10×). Results are means ± SD; *** $p \leq 0.001$.
